# The Role of Calcineurin/NFAT in SFRP2 Induced Angiogenesis—A Rationale for Breast Cancer Treatment with the Calcineurin Inhibitor Tacrolimus

**DOI:** 10.1371/journal.pone.0020412

**Published:** 2011-06-03

**Authors:** Sharareh Siamakpour-Reihani, Joseph Caster, Desh Bandhu Nepal, Andrew Courtwright, Eleanor Hilliard, Jerry Usary, David Ketelsen, David Darr, Xiang Jun Shen, Cam Patterson, Nancy Klauber-DeMore

**Affiliations:** 1 Lineberger Comprehensive Cancer Center, University of North Carolina at Chapel Hill, Chapel Hill, North Carolina, United States of America; 2 Department of Surgery, University of North Carolina at Chapel Hill, Chapel Hill, North Carolina, United States of America; 3 UNC McAllister Heart Institute, University of North Carolina at Chapel Hill, Chapel Hill, North Carolina, United States of America; National Cancer Institute, United States of America

## Abstract

Tacrolimus (FK506) is an immunosuppressive drug that binds to the immunophilin FKBPB12. The FK506-FKBP12 complex associates with calcineurin and inhibits its phosphatase activity, resulting in inhibition of nuclear translocation of nuclear factor of activated T-cells (NFAT). There is increasing data supporting a critical role of NFAT in mediating angiogenic responses stimulated by both vascular endothelial growth factor (VEGF) and a novel angiogenesis factor, secreted frizzled-related protein 2 (SFRP2). Since both VEGF and SFRP2 are expressed in breast carcinomas, we hypothesized that tacrolimus would inhibit breast carcinoma growth. Using IHC (IHC) with antibodies to FKBP12 on breast carcinomas we found that FKBP12 localizes to breast tumor vasculature. Treatment of MMTV-neu transgenic mice with tacrolimus (3 mg/kg i.p. daily) (n = 19) resulted in a 73% reduction in the growth rate for tacrolimus treated mice compared to control (n = 15), p = 0.003; which was associated with an 82% reduction in tumor microvascular density (p<0.001) by IHC. Tacrolimus (1 µM) inhibited SFRP2 induced endothelial tube formation by 71% (p = 0.005) and inhibited VEGF induced endothelial tube formation by 67% (p = 0.004). To show that NFATc3 is required for SFRP2 stimulated angiogenesis, NFATc3 was silenced with shRNA in endothelial cells. Sham transfected cells responded to SFRP2 stimulation in a tube formation assay with an increase in the number of branch points (p<0.003), however, cells transfected with shRNA to NFATc3 showed no increase in tube formation in response to SFRP2. This demonstrates that NFATc3 is required for SFRP2 induced tube formation, and tacrolimus inhibits angiogenesis *in vitro* and breast carcinoma growth *in vivo*. This provides a rationale for examining the therapeutic potential of tacrolimus at inhibiting breast carcinoma growth in humans.

## Introduction

Tumor angiogenesis is regulated by multiple proangiogenic factors, of which the most widely studied is vascular endothelial growth factor (VEGF). One of the pathways through which VEGF stimulates angiogenesis is through activation of calcineurin/nuclear factor of activated T-cells (NFAT) signaling [1]–[3]. Recent work has identified a novel angiogenesis factor, secreted frizzled-related protein 2 (SFRP2), which is expressed in the vasculature of a wide variety of tumors including human breast carcinoma [4], [5]. SFRP2 stimulates angiogenesis in the mouse Matrigel plug assay, induces endothelial cell migration and tube formation *in vitro*, and protects against hypoxia-induced apoptosis in endothelial cells [5].

SFRP2 belongs to a large family of secreted frizzle-related proteins (SFRPs) which are related to the Wnt signaling cascade. Wnt proteins have been grouped into two classes – canonical and noncanonical – on the basis of their activity in cell lines or *in vivo* assays. The core of the canonical Wnt pathway is the stability of beta catenin [6]. SFRPs have been regarded as inhibitors of the canonical Wnt-beta catenin pathway [6], while recent studies have shown that SFRP2 can increase nuclear beta catenin levels [7]–[10]. In contrast we previously found that treatment of endothelial cells with SFRP2 (at angiogenic doses) resulted in no change in nuclear beta catenin levels in endothelial cells [5], suggesting that SFRP2 does not stimulate angiogenesis through inhibition or activation of the Wnt/beta catenin pathway.

Noncanonical Wnts activate other signaling pathways, such as the Wnt/Ca^2+^ pathway [11]. The Wnt/Ca^2+^ pathway is a beta catenin-independent pathway for which signaling is mediated through transient increases in cytoplasmic free calcium which activates the phosphatase calcineurin. Activated calcineurin dephosphorylates NFAT, which then translocates to from the cytoplasm to the nucleus [12]. NFAT is a multigene family containing five members: NFAT (NFATc1-c5). Except for NFAT5, which is activated in response to osmotic stress [13], all NFAT family members are regulated by the calcium-activated protein phosphatase calcineurin and exist as transcriptionally inactive, cytosolic phosphoproteins [12]. There is increasing data supporting a critical role of NFAT in mediating angiogenic responses [1]–[3]. Importantly, NFAT activation was identified as a critical component of VEGF-induced angiogenesis and linked to the induction of cyclooxygenase-2 [14], which is also a critical player in angiogenesis. Our data suggested that NFAT may also mediate SFRP2 induced angiogenesis, as treatment of endothelial cells with SFRP2 resulted in an increase in nuclear NFATc3 [5].

In this study we further elucidate the role of both beta catenin and NFATc3 in SFRP2 mediated angiogenesis through RNA silencing, which confirms that NFATc3 is required for SFRP2 induced angiogenesis, while beta catenin is not. Thus, targeting NFAT with a calcineurin inhibitor may be a therapeutic strategy to inhibit both VEGF and SFRP2 induced angiogenesis. Tacrolimus (FK506) is an immunosuppressive drug that binds to the immunophlin FKBPB12, and the FK506-FKBP12 complex associates with calcineurin and inhibits its phosphatase activity, resulting in inhibition of nuclear translocation of NFAT [1]. Tacrolimus is FDA approved for the prevention of organ transplant rejection and acts by inhibiting NFAT in lymphocytes [15]. Since FKBP12 has been reported to be expressed in benign and malignant vascular endothelium [16], we hypothesize that FKBP12 is expressed in breast tumor endothelium, allowing tacrolimus to inhibit breast tumor angiogenesis and tumor growth.

## Materials and Methods

### Treatment of MMTV-neu transgenic mice with tacrolimus in vivo

This study was carried out in strict accordance with the recommendations in the Guide for the Care and Use of Laboratory Animals of the National Institutes of Health. The protocol was approved by the Committee on the Ethics of Animal Experiments of the University of North Carolina at Chapel Hill, IACUC ID# 09-134.0. We had previously shown that treatment with tacrolimus 3 mg/kg/day intraperitoneal (i.p.) was effective at suppressing the growth of SVR angiosarcoma tumor in nude mice as compared with control by 46% without signs of toxicity [5]. Using an FDA Oncology Calculator, we calculated this dose to be equivalent to the human dose of 0.24 mg/kg/day [5], which is the dose that is used to prevent human liver transplant rejection. To evaluate whether tacrolimus would inhibit the growth rate of breast tumors *in vivo*, we studied the efficacy of tacrolimus on the growth of MMTV-neu tumors in transgenic mice. Female FVB/N-Tg(MMTVneu) 202Mul/J mice were purchased from the Jackson Laboratory (Bar Harbor, ME, Stock Number 002376). The experimental female mice were bred in order to speed the onset of tumorigenesis and were housed and inspected for tumor development twice weekly. All animal work was conducted in UNC DLAM animal facilities under an approved IACUC protocol. Once tumors were detected by palpation or visual inspection, tumor volume was documented using calipers and tumor volume was calculated using the following formula: (shortest diameter)^2^×(longest diameter)×0.52. When tumors reached 100–300 mm^3^, mice were treated with either tacrolimus 3 mg/kg/day i.p. in 20% Intralipid (Baxter, Deerfield, IL) or control (which consisted of no treatment n = 9 or 20% Intralipid i.p. n = 6). All tumors that were present from day 1 were measured. Mice used in this study developed either one or two mammary tumors. Tumor growth (assessed as percent change in tumor volume over initial tumor volume) was calculated using the formula: [(final volume−initial volume)/initial volume×100].

### Antibodies

The following antibodies were purchased from Santa Cruz Biotechnology, Inc., Santa Cruz, CA: FKBP12 (sc-28814), NFTAc3 (sc- 8405), SFRP2 (sc-13940), and β-catenin (sc-59893). The loading control, TATA binding protein TBP antibody (ab818), was purchased from Abcam, Inc. (Cambridge, MA). CD31 primary antibody was purchased from NeoMarkers (Fremont, CA). Secondary antibodies were purchased from GE Healthcare Bio-Sciences Corp. (Piscataway, NJ): ECL anti-mouse IgG, HRP-linked whole antibody (NA931) and ECL anti-rabbit IgG, HRP-linked whole antibody (NA934).

### Immunohistochemistry

#### CD31 staining in mouse breast tumors

To show whether the reduced growth rate of tacrolimus treated tumors correlates with a decrease in tumor angiogenesis, we sectioned 4 control tumors from mice treated with 20% intralipid and 4 tumors from mice treated with tacrolimus. Breast tumors were sectioned at 5 µM onto Superfrost plus slides. Slides were dewaxed by immersing in xylene three times for 5 minutes each. Slides were hydrated in 100% ETOH, 95% ETOH, 70% ETOH, and 50% ETOH for 3 minutes each. Slides were quenched in 3% H2O2 (DakoCytomation, LSAB2 HRP Kit, Carpinteria, CA) for 10 minutes, and then PBS for 3 minutes. Citra buffer (BioGenex, San Ramon, CA) was warmed in a 60°C oven and slides were immersed in citra buffer at 100°C in a rice steamer for 30 minutes. Slides were rinsed in PBS for 3 minutes and then marked with a PAP pen. Goat serum (Pierce Biotechnology, Rockford, IL) block (3 mg/ml) was applied for 10 minutes at room temperature. Slides were rinsed twice in PBS for 3 minutes then CD31 primary antibody (250 µl at 1∶100 dilution) was applied and slides were placed in a covered box in a 4°C cold room overnight. Slides were then rinsed in PBS for 3 minutes, and 1–2 drops of biotinylated secondary antibody (Ultravision Detection System, LabVision Corp, Fremont, CA) was added to each slide for 20 minutes. Slides were rinsed twice in PBS for 3 minutes and 1–2 drops of streptavidin-HRP (Ultravision Detection System, LabVision Corp, Fremont, CA) was applied for 20 minutes. One-two drops of DAB complex (Ultravision Detection System, LabVision Corp, Fremont, CA) was applied to each slide for approximately 10 minutes. Slides were rinsed twice in distilled water for 3 minutes each and counterstained with trypan blue (Sigma, St Louis, MO) for 5 minutes. Slides were rinsed in PBS, dehydrated through graded alcohol and xylene, and Cytoseal XYL (Richard-Allan, Kalamazoo, MI) and cover slides were applied. A negative control without primary antibody was performed for all experiments. Slides were evaluated for the presence of CD31 staining in tumor endothelium.Tumor vascularity was quantified as described previously to identify the number of microvessels/unit area (×200) [17]–[20]. The mean of three fields judged to have the greatest numbers of microvessels was used for comparison between control and tacrolimus treated tumors.

#### FKBP12 staining in human breast tumors

Research involving archival human samples was approved by the IRB at the University of North Carolina at Chapel Hill on an IRB approved protocol # 05-2442. A waiver for obtaining consent was obtained because the research involved only existing human biological specimens. To evaluate whether the binding protein of tacrolimus, FKBP12, is present in the vasculature of human breast tumors, we stained paraffin-embedded human breast tumors and mouse MMTV-neu breast carcinomas (on an IACUC approved protocol) with a polyclonal antibody to FKBP12. IHC was performed as described above, accept the primary antibody was FKBP12 antibody (100 µl–200 µl at 1∶200 dilution). A negative control without primary antibody was performed for all experiments. Slides were evaluated for the presence of FKBP12 staining in tumor endothelium.

### Cell culture

2H11 mouse endothelial cells (American Type Culture Collection ATCC®, Manassas, VA) were cultured in Dulbecco's modified Eagle's medium (DMEM) with 4.5 g/L glucose (Sigma-Aldrich, St. Louis, MO) with 10% fetal bovine serum (FBS) (Sigma-Aldrich, St. Louis, MO). MMTV-neu breast tumor cells [21] (a gift from Dr. John Serody, UNC-Chapel Hill) were cultured in GIBCO® RPMI 1640 media (Invitrogen, Carlsbad, CA) 20%FBS, 2 mM L-glutamine, 12 mM, HEPES, 0.1 mM NEAA, 1 mM Sodium pyruvate, 1% Pen Strep, 50 uM 2-ME, 0.2 U/ml Regular insulin.

### siRNA to beta catenin in endothelial cells

To prove that SFRP2 does not stimulate tube formation via the beta catenin pathway, we silenced beta catenin in endothelial cells and evaluated their ability to respond to SFRP2 treatment in a tube formation assay. 2H11 mouse endothelial cells were transfected with siRNA to beta catenin and control nonsilencing siRNA. The siRNA for beta catenin, which is a pool of 4 target- specific 21 nt siRNAs (beta catenin siRNA (m): sc-29210, Santa Cruz Biotechnology, Santa Cruz, CA), and nonsilencing siRNA (sc-36869, Santa Cruz Biotechnology) were used in 100 pmol/ml (100 nM) concentration. The 2H11 cells were maintained in DMEM with 10% FBS and were transfected with siRNA to beta catenin or the control siRNA using Lipofectamine™ RNAiMAX transfection reagent (Invitrogen, Carlsbad, CA) according to the manufacturer's protocol. The transfection efficiency was determined using siGLO Green Transfection indicator (Dharmacon, Inc. cat# D0016300105) according to the manufacturer's instruction.

To verify the knockdown of beta catenin, cells were harvested 72 hrs post transfection for protein analysis by Western blot. Nuclear extracts using NE-PER nuclear and cytoplasmic extraction reagent were prepared as described in the manufacturer's manual (Pierce Biotechnology, Rockford, IL). Nuclear fractions were confirmed on Western blot using the loading control, TATA binding protein TBP antibodies, which is a nuclear marker. Protein concentration was measured using Bio-Rad Protein Assay at OD_595_ (Bio-Rad Laboratories). Equal amounts of protein (20 µg) were loaded onto SDS-PAGE gels. Proteins were transferred to Polyvinylidene Difluoride membrane (PVDF), and Western blotting was carried out using a primary antibody to beta catenin, with horseradish peroxidase (HRP)-conjugated IgG as the secondary antibody. The ECL Advance substrate was used for visualization (GE Healthcare Bio-Sciences).

### shRNA to NFATc3 in endothelial cells

To evaluate the functional results of silencing of NFATc3 in SFRP2 stimulated endothelial cell tube formation, we used shRNAmir to NFATc3 (Open Biosystems/Thermo Scientific). The sh-RNA plasmids were supplied in E.coli cells from Open Biosystems. They were grown overnight at 37°C with shaking in 100 ug/ml ampicillin/LB broth. The culture was spun down and plasmids were extracted to be used in transfecting the 2H11 cells. Three different shRNAmir constructs to NFATc3 from Open Biosystems were tested: a) RMM4431-99213393 Mouse GIPZ lentiviral shRNAmir individual clone V2LMM_110117, b) RMM4431-98727337 Mouse GIPZ lentiviral shRNAmir individual clone V2LMM_110113 (resulting in the best down regulation of NFATc3), c) RMM4431-98750613 Mouse GIPZ lentiviral shRNAmir individual clone V2LMM_110112, RHS4430-98525659 Human GIPZ lentiviral shRNAmir individual clone V2LHS_161372. Control shRNAmir constructs were GADPH-pGIPZ and non silencing- pGIPZ construct purchased from Open Biosystems.

In order to establish stable cell lines expressing the shRNA to NFATc3 we first generated a puromycin kill curve for the 2H11 endothelial cells. The minimum concentration of puromycin required to kill non-transfected 2H11 cells was 4 ug/ml. The 2H11 cells were then seeded in 6 well plates 24 hours prior to transfection using Lipofectamine™ LTX Transfection reagent (Invitrogen) according to the manufacturer's protocol. Forty-eight hours post transduction the DMEM 10% FBS media was replaced with full growth selective media (containing 4 ug/ml Puromycin) (Mediatech, Inc., Manassas, VA) into the appropriate wells. The selective media was changed every two-three days. After selection the cells were kept in full growth media containing 2 ug/ml puromycin. Western blot analysis on nuclear extracts using NE-PER nuclear and cytoplasmic extraction reagent (Pierce Biotechnology) was done to evaluate the degree of NFATc3 silencing. This indicated the best results for knock down of NFATc3 was obtained with the RMM4431-98727337 Mouse GIPZ lentiviral shRNAmir construct.

### Western blot analyses for NFATc3 in tacrolimus treated endothelial cells

2H11 mouse endothelial cells were grown to 80–90% confluence in DMEM with 10% FBS. The 2H11 cells were serum starved in DMEM with 2% FBS overnight. The following day the media was changed to DMEM with 5% FBS and supplements. Control cells received 1.5% DMSO; SFRP2-treated cells received mouse recombinant SFRP2 7 nM with DMSO 1.5%; and tacrolimus treated cells received tacrolimus (10 uM), in 1.5% DMSO with and without mouse recombinant SFRP2 (7 nM). Cells were incubated for one hour with the SFRP2/tacrolimus treatment. Nuclear and cytoplasmic proteins were extracted by using NE-PER nuclear and cytoplasmic extraction reagent. For experiments extracting whole-cell lysates, the M-PER Mammalian Protein Extract reagent (Pierce Biotechnology) was used as described in the manufacturer's manual. Western blot was performed as described above.

### Endothelial tube formation assay in vitro

A Matrigel tube formation assay was used to evaluate whether silencing beta catenin or NFATC3 in endothelial cells augments *de novo* or SFRP2 induced angiogenesis. ECMatrix (Chemicon, Temecula, CA) was thawed, diluted, and solidified in a 96 well plate according to the manufacturer's instructions. 2H11 endothelial cells, sham transfected 2H11 cells, siRNA beta catenin transfected or shRNA NFATc3 transfected cells were kept in appropriate media. 48 hrs post-transfection the media was changed to DMEM with 2% FBS. 72 hrs post-transfection the cells were seeded onto the matrix at 5,000 cells/well in 150 µl of DMEM with 5% FBS and supplements and incubated with and without mouse recombinant SFRP2 (R&D Systems, Inc., Minneapolis, MN) (7 nM) at 37°C, 5% CO2 for 6 hours. Wells were photographed and tube formation was quantified by counting the number of branch points.

To evaluate whether tacrolimus inhibits endothelial tube formation *in vitro*, we evaluated the effect of tacrolimus on SFRP2 and VEGF induced tube formation. ECMatrix was thawed, diluted, and solidified in a 96 well plate. 2H11 endothelial cells were serum starved in DMEM with 2% FBS overnight, and then seeded onto the matrix at 5,000 cells/well in 150 µl of DMEM with 5% FBS and supplements. Control cells received 1.5% DMSO; SFRP2-treated cells received mouse recombinant SFRP2 7 nM with DMSO 1.5%; and tacrolimus treated cells received mouse recombinant SFRP2 7 nM with tacrolimus (LC Laboratories, Manassas, VA) (10 uM, 1 uM, and 0.1 uM in 1.5% DMSO). The plates were returned to 37°C, 5% CO2 for 6 hours. Wells were photographed and tube formation was quantified by counting the number of branch points. The experiment was repeated using human recombinant VEGF (60 ng/ml) (Cat# 293-VE/CF, R&D Systems, Minneapolis, MN) in place of SFRP2.

### Endothelial cell and breast tumor cell migration assay

The migration properties of tacrolimus on MMTV-neu breast tumor cells and 2H11 endothelial cells were determined using a scratch wound assay. MMTV-neu cells were seeded at a concentration of 80,000 cells/well in 96 well-plates in a complete media (RPMI-1640, 20%FBS, 2 mM L-glutamine, 12 mM HEPES, 0.1 mM NEAA, 1 mM Sodium pyruvate, 1% Pen Strep, 50 uM 2-ME, 0.2 U/ml Regular insulin). After 24 hours, cells were starved in RPMI with 1% FBS. 2H11 endothelial cells were seeded at a concentration of 10,000 cells/well in a 96 well plate in DMEM with 10%FBS and mouse recombinant SFRP2 (700 pM). After 24 hours, 2H11 endothelial cells were starved in DMEM with 2% FBS. For both cell lines, 20 hours into starvation a scratch wound was made using a 1 mL pipette tip and the media was changed to DMEM with 5% FBS. Cells were treated with 1.5% DMSO (control), or tacrolimus at a concentration of 10 uM, 1 uM, and 0.1 uM in 1.5% DMSO. The distance of the wound was measured with an ocular micrometer at 0, 12, 18 and 24 hr into treatment.

### Statistical analyses

Statistical differences between treated and control were calculated using a two-tailed student's T-test, with a p≤0.05 being significant. Results are expressed as means ± standard error of the mean (SEM).

## Results

### Tacrolimus inhibits breast tumor growth in vivo

**Figure 1:**
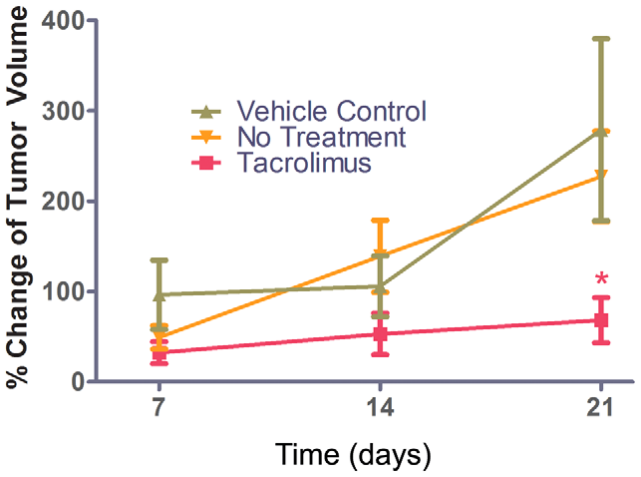
Tacrolimus inhibited the growth rate of MMTV-neu transgenic mouse tumors. MMTV-neu transgenic mice were treated with tacrolimus 3 mg/kg/day i.p., no treatment control, 20% intralipid i.p. control beginning when tumors became palpable, and was continued for 21 days. Tumor volumes were measured on day 7, 14 and 21, and the growth rate (percent change in tumor volume per day) was compared between control (no treatment), control (20% intralipid) and tacrolimus treated groups. There was no statistically significant difference between control (untreated) and control (20% intralipid) groups, and therefore the controls were combined for analyses. At 21 days there was a 73% reduction in the growth rate for tacrolimus treated mice compared to no treatment (n = 19 tacrolimus treated, n = 15 control, *p = 0.003). Tacrolimus treated mice were significantly different from control (no treatment, p = 0.01, and control 20% intralipid, p = 0.01). There was no weight loss or lethargy in the tacrolimus-treated mice or the 20% intralipid treated mice.

To evaluate whether tacrolimus inhibits breast carcinoma growth, we compared MMTV-neu transgenic mice treated with tacrolimus in 20% intralipid (n = 19) at 3 mg/kg/day i.p. for 21 days to control mice that received no treatment (n = 9) or control with 20% intralipid i.p. (n = 6). There was no statistically significant difference in the growth rate between control with no treatment (227±51) and control with 20% intralipid (279±101, p = 0.6), demonstrating that the control vehicle did not affect tumor growth. Therefore the control groups were combined for analyses. The mean growth rate (% change in tumor volume) after 21 days was 68±25 in tacrolimus treated mice, compared to the combined controls (n = 15) 248±48, which is a reduction of 73% (p = 0.003, Fig. 1). When tacrolimus is compared to the no treatment controls the p = 0.01, and to the 20% intralipid controls the p = 0.01. There were no signs of toxicity (i.e., diarrhea, infection, lethargy, or weight loss) after treatment. This demonstrates that tacrolimus inhibits the growth rate of breast carcinoma *in vivo*.

### Microvessel density is decreased in tacrolimus treated tumors

**Figure 2:**
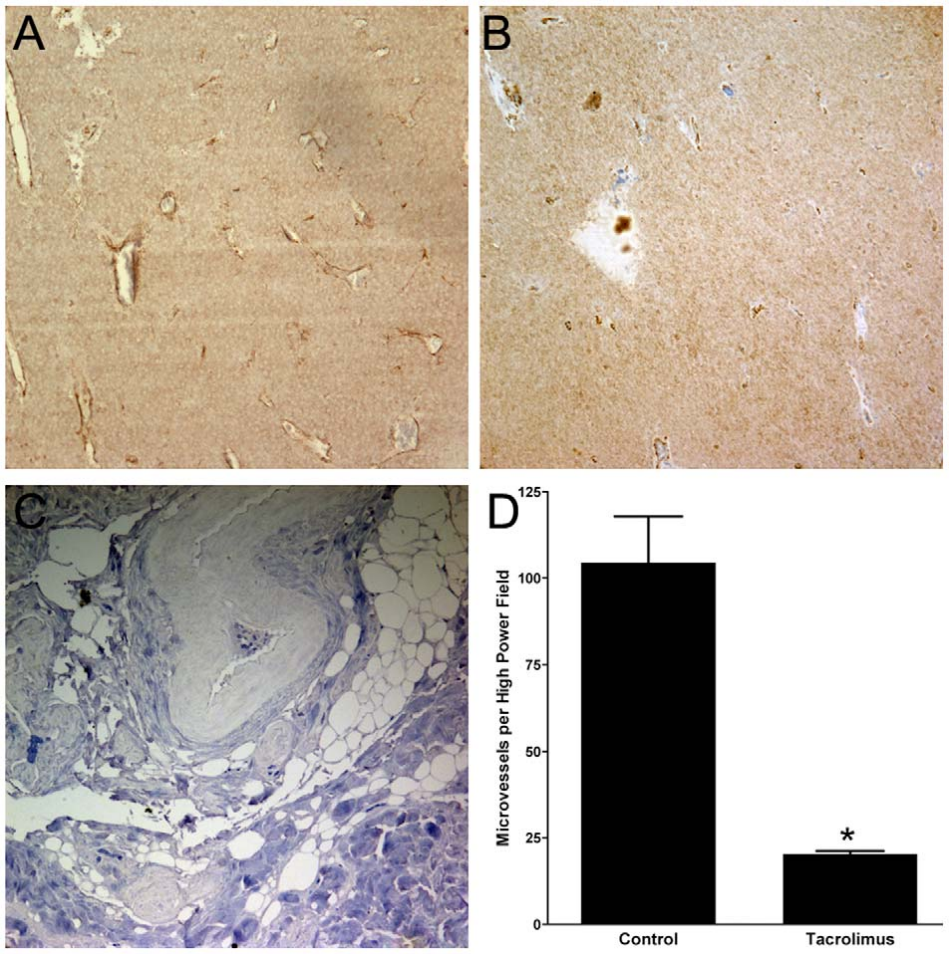
Tacrolimus reduces tumor vascularity. MMTVneu tumors from 4 control 20% intralipid mice and 4 tacrolimus treated mice were resected and embedded in paraffin. Immunohistochemistry with antibody to factor VIII was performed as described in “Material and Methods”. Microvessel density was determined by counting the number of microvessels in 3 high-power fields at 200×. A, D) The mean number of microvessels per high power field in control tumors was 104±13. B, D) The mean number of microvessels per high power field in tacrolimus treated tumors was 20±1 (p<0.001). This shows that tacrolimus reduces angiogenesis *in vivo*. C) Negative control human breast tumor without primary antibody shows no staining.

To show whether decreased tumor growth in tacrolimus treated mice correlates with a decrease in tumor angiogenesis, we performed IHC on paraffin embedded tumors from control (20% intralipid, n = 4) mice and tacrolimus treated mice (n = 4). The number of microvessels per high power field for control tumors was 104±13, compared to tacrolimus tumors 20±1, p<0.001 (Fig. 2), indicating that tacrolimus decreased tumor angiogenesis *in vivo*.

### FKBP12, the binding partner of tacrolimus, is expressed in human and mouse breast tumor endothelium by immunohistochemistry

We evaluated the vascular staining of FKBP12 in paraffin embedded human and mouse breast tumors using IHC with antibodies to FKBP12, and found that FKBP12 localized to human invasive ductal carcinomas in 8 of 11 tumors; and mouse MMTV-neu breast tumor endothelium in 8 of 8 tumors (Figure S1). Since the binding partner for tacrolimus is expressed in human breast tumor vasculature, there is the potential that tacrolimus could bind to FKBP12 and inhibit angiogenesis in human breast tumors.

### Beta catenin is not required for SFRP2 induced endothelial tube formation

**Figure 3:**
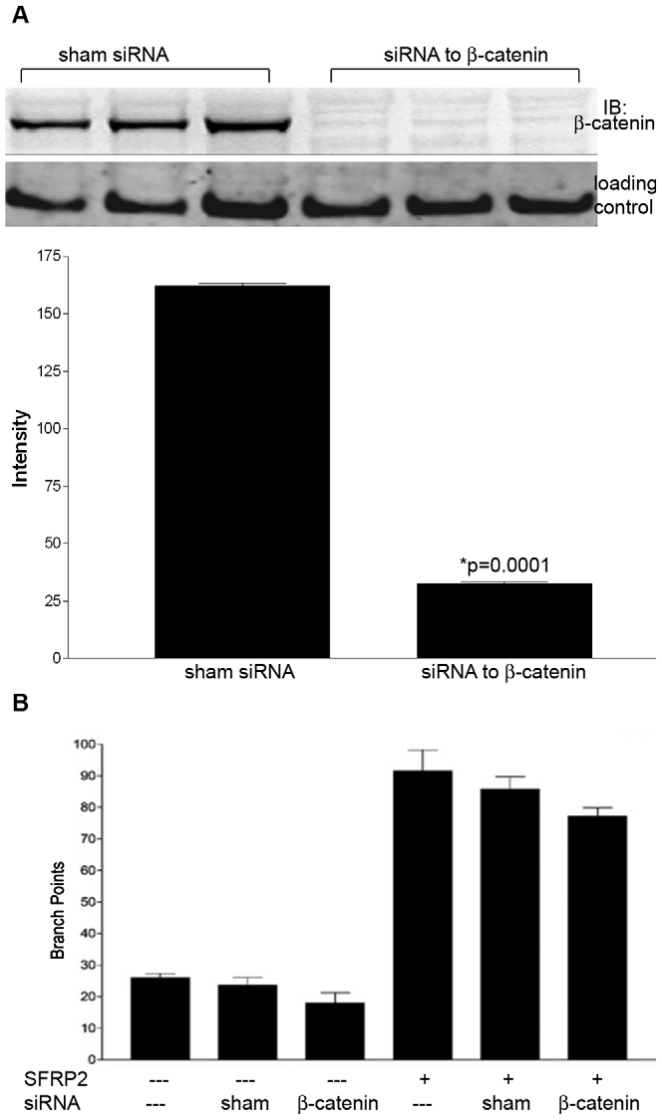
Beta catenin was not required for SFRP2 induced endothelial tube formation: 2H11 endothelial cells were transfected with siRNA to beta catenin or sham transfected. A) Western blot results of siRNA to beta catenin showed silencing of beta catenin by 80%. The loading control was TATA binding protein TBP antibodies (a nuclear marker). B) 2H11 endothelial cell tube formation assay. Cells were plated in Matrigel as described in “Material and Methods”. After 6 hours the number of branch points were counted. There was no significant decrease in *de novo* or SFRP2 (7 nm) induced tube formation following the silencing of beta catenin. Full-length blots/gels are presented in Figure S2A.

To show whether beta catenin is involved in SFRP2-stimulated angiogenesis, beta catenin was silenced in 2H11 endothelial cells with siRNA and their ability to undergo tube formation was compared to sham-transfected cells. Western blot analyses of 2H11 cells transfected with siRNA to beta catenin demonstrated 80% knockdown of beta catenin (Fig. 3A, Figure S2). Some studies have shown that SFRP2 is an inhibitor of beta catenin [6]; if the mechanism through which SFRP2 stimulated tube formation is through inhibition of beta catenin signaling, then we would expect that silencing beta catenin in endothelial cells would increase tube formation. However, we found no difference in *de novo* tube formation between sham transfected cells and siRNA beta catenin transfected cells (Fig. 3B). Other studies have shown that SFRP2 can increase nuclear beta catenin levels [7]–[10]; if the mechanism through which SFRP2 stimulated tube formation was through activation of beta catenin signaling, then we would expect that silencing beta catenin in endothelial cells would block SFRP2 induced tube formation. However, there was no difference in tube formation between sham-transfected cells stimulated with SFRP2 (7 nM) and siRNA beta catenin transfected cells stimulated with SFRP2 (7 nM) [Fig. 3B]. This demonstrates that beta catenin is not required for SFRP2 induced tube formation.

### NFATc3 is required for SFRP2 induced endothelial tube branching

**Figure 4:**
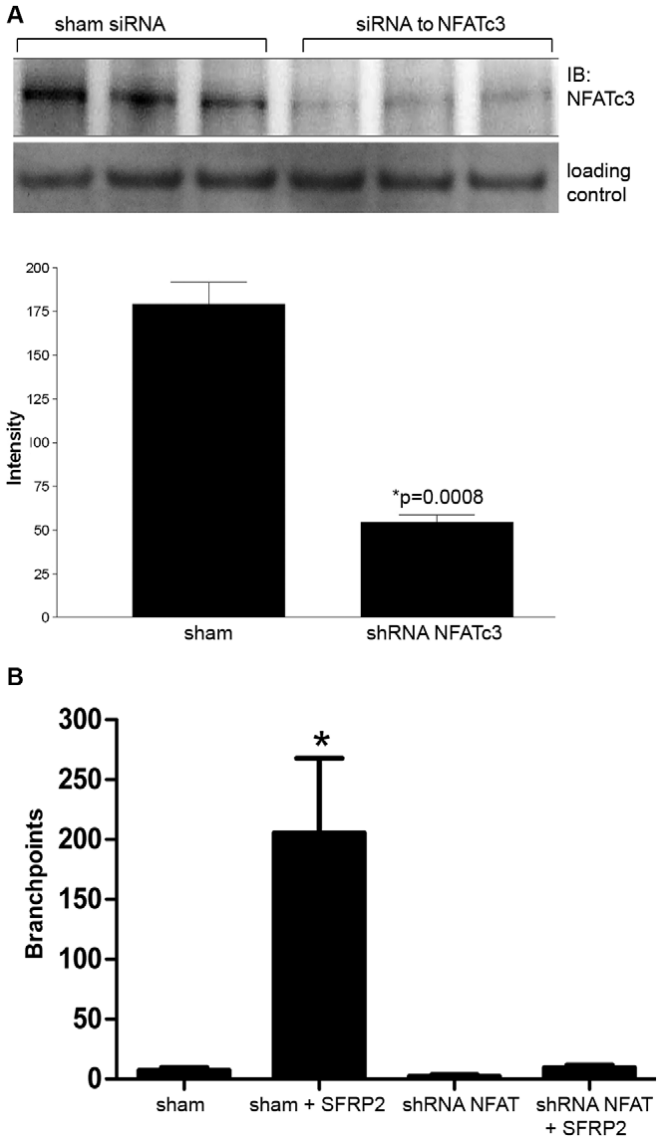
NFATc3 was required for SFRP2 stimulated tube formation. A) ShRNA to SFRP2 in 2H11 endothelial cells showed 69% reduction in NFATc3 level by Western blot. The loading control was TATA binding protein TBP antibodies (a nuclear marker). B) Sham transfected 2H11 cells increased tube formation in response to SFRP2 (7 nM) (n = 3 for all groups, p<0.01), which was not seen in shRNA to NFATc3 transfected cells. Full-length blots/gels are presented in Supplemental Figure S2B. Pictures of sham transfected cells and shRNA to NFATc3 transfected cells (both stimulated with SFRP2 (7 nM)) are in Figure S3.

We have reported that endothelial cells treated with SFRP2 have an increase in nuclear NFATc3, and inhibition of NFAT in endothelial cells with the calcineurin inhibitor tacrolimus inhibits SFRP2 stimulated tube formation [5]. To definitively show whether NFATc3 is required for SFRP2 stimulated angiogenesis, we established a stable 2H11 endothelial cell line with shRNA to NFATc3, and Western blot analysis demonstrated that NFATc3 protein is decreased in shRNA-NFATc3 transfected cells by 69% compared to sham-transfected controls (Fig. 4A, Supplemental Fig. S2B). Cells were then seeded for a 6 hour tube formation assay. Sham transfected cells responded to SFRP2 stimulation with a statistically significant increase in the number of branch points (p<0.01) (Fig. 4B, Figure S3A). However, 2H11 cells transfected with shRNA to NFATc3 showed no increase in tube formation in response to SFRP2 stimulation (Fig. 4B, Figure S3B). This shows that NFATc3 is required for SFRP2 mediated endothelial tube formation.

### Tacrolimus inhibits SFRP2 activation of NFATc3 in endothelial cells

**Figure 5:**
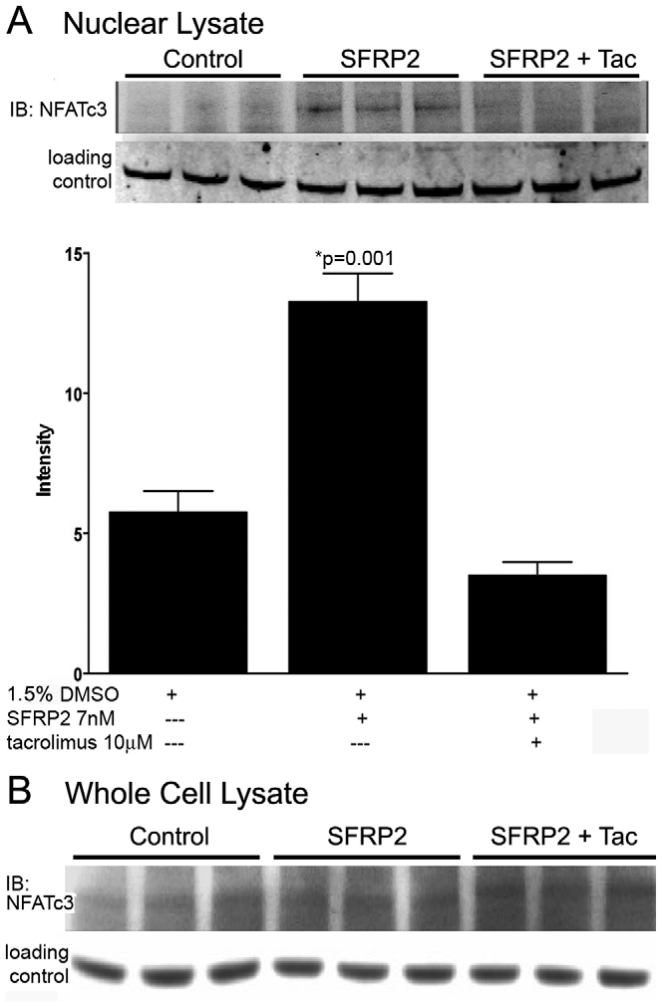
Tacrolimus decreased SFRP2 induced nuclear NFATc3 in 2H11 endothelial cells. A) 2H11 cells were treated with control (1.5% DMSO), mouse recombinant SFRP2 (7 nM)+1.5% DMSO; or SFRP2 7(nM)+tacrolimus 10 µM in 1.5% DMSO for 1 hour, and nuclear protein lysates were collected and analyzed by Western blot analyses probing for NFATc3 as described in “Material and Methods”. The loading control was TATA binding protein TBP antibodies (a nuclear marker). SFRP2 increased nuclear NFATc3 compared to control cells (p = 0.003). Full-length blots/gels are presented in Figure S2C. B) The experiment was repeated as above except that whole cell lysates rather than nuclear lysates were extracted and analyzed by Western blot analyses probing for NFATc3. The loading control was beta-tubulin. Tacrolimus did not inhibit total NFATc3 protein. Taken together this shows that tacrolimus inhibits SFRP2 induced NFATc3 nuclear translocation but not total protein levels.

To evaluate the role of tacrolimus on the non-canonical Wnt/Ca^++^ pathway in SFRP2 induced angiogenesis, we compared nuclear dephosphorylated NFAT protein levels in control, SFRP2-treated endothelial cells, and SFRP2 and tacrolimus treated cells. After one hour of treatment of 2H11 endothelial cells with mouse recombinant SFRP2 (7 nM), nuclear NFATc3 was increased 2 fold (p = 0.003) (Fig. 5A). However, when tacrolimus (10 uM) was added to SFRP2 (7 nM) treatment, there was no increase in nuclear NFATc3 protein levels (Fig. 5A, Figure S2C). To evaluate whether the reduction in nuclear NFAT induced by tacrolimus is from nuclear translocation and not simply reduced expression of NFAT, we evaluated the effect of tacrolimus treatment on NFATc3 protein in whole cell lysates. There was no reduction of NFATc3 with tacrolimus treatment (Figure 5B). This shows that tacrolimus inhibits SFRP2 induced NFATc3 translocation in endothelial cells without reducing total NFATc3 protein.

### Tacrolimus inhibits SFRP2 and VEGF -Induced Endothelial Cell Tube Formation

**Figure 6:**
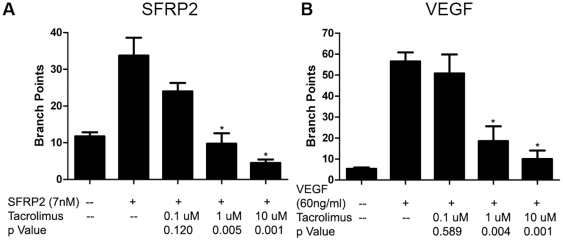
Tacrolimus inhibited SFRP2 and VEGF stimulated endothelial tube formation *in vitro*. 2H11 endothelial cells were plated in Matrigel as described in “Material and Methods”. A) SFRP2 induced endothelial tube formation after 6 hours, which was inhibited by tacrolimus in a concentration dependent manner. B) VEGF induced endothelial tube formation after 6 hours, which was inhibited by tacrolimus in a concentration dependent manner.

We previously showed that tacrolimus can inhibit SFRP2-mediated tube formation *in vitro* [5]. In this study we explored whether tacrolimus would also inhibit VEGF induced tube formation, as VEGF has been reported to activate the calcineurin/NFAT pathway [1]–[3]. 2H11 endothelial cells were stimulated with either SFRP2 or VEGF +/− tacrolimus to determine if tacrolimus inhibited tube formation stimulated by both mitogens. Tacrolimus (1 µM) inhibited SFRP2 induced 2H11 tube formation by 71% (p = 0.005, Fig. 6A), and tube formation was inhibited in a concentration dependent manner. Tacrolimus (1 µM) also inhibited VEGF induced 2H11 tube formation by 67% (p = 0.004, Fig. 6B). Tacrolimus was not cytotoxic to 2H11 cells, as less than 5% of tacrolimus-treated cells took up trypan blue dye (data not shown). This shows that tacrolimus inhibits both SFRP2 and VEGF induced tube formation and therefore could potentially block angiogenesis stimulated by both mitogens.

### Tacrolimus inhibits breast tumor and endothelial cell migration

**Figure 7:**
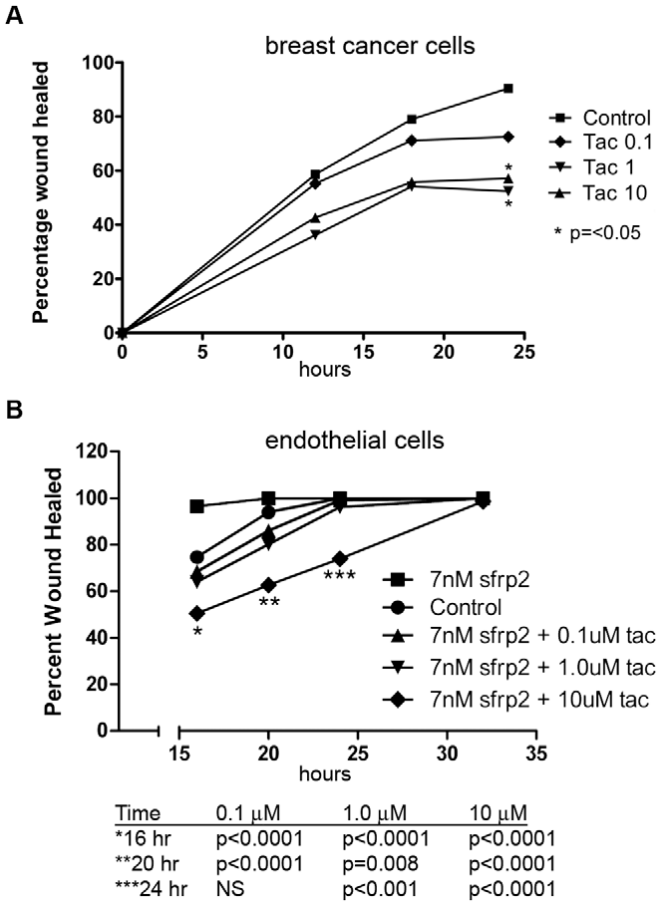
Tacrolimus inhibited breast cancer and endothelial cell migration in a scratch wound migration assay. A) MMTV-neu breast cancer cells were plated in a scratch wound assay as described in “Material and Methods”. A wound was formed with a 1 mm pipette tip, and tacrolimus or control was added to the wells. Migration was measured at various time points with an ocular micrometer. Tacrolimus statistically significantly inhibited MMTV-neu breast tumor migration at 1 and 10 µM (p<0.05). B) 2H11 cells were plated in a tube formation assay without SFRP2 (Control 1.5% DMSO), with SFRP2 7 nM and 1.5% DMSO, or with SFRP2 (7 nM)+tacrolimus (0.1–10 µM in 1.5% DMSO). SFRP2 induced endothelial cell migration compared to control, which was statistically significantly inhibited by tacrolimus at 0.1, 1 and 10 µM.

The migration properties of tacrolimus on 2H11 endothelial cells and MMTV-neu breast carcinoma cells were evaluated using a scratch wound assay. Tacrolimus (1 uM) inhibited the migration of MMTV-neu cell migration at 24 hours by 45% (p = 0.04, Fig. 7A), and inhibited the migration of SFRP2 stimulated 2H11 cells at 20 hours by 20% (P = 0.008, Fig. 7B). This shows that tacrolimus has a direct effect on breast tumor cells in addition to its antiangiogenic effect.

## Discussion

This study provides further evidence for a novel pathway of SFRP2 signaling in endothelial cells through activation of NFAT, a pathway shared with other angiogenesis stimulators such as VEGF. Although SFRPs have been regarded as inhibitors of the canonical Wnt-beta catenin pathway [6], four recent studies have shown that SFRP2 can act as a Wnt *agonist rather than an antagonist*. SFRP2 has been found to increase nuclear beta catenin levels in: 1) cardiomyocytes exposed to hypoxia and treated with mouse recombinant SFRP2 [7], 2) MCF7 cells with stable transfectants of SFRP2 [8], 3) mammary canine tumors with increased expression of SFRP2 [9], and hypoxic adipose tissue derived stem cells [10]. In contrast, we previously found that there was no change in nuclear beta catenin in endothelial cells stimulated with SFRP2 at doses that induced angiogenesis *in vitro*, although we did see an increase in nuclear beta catenin at a four-fold higher dose. In this study we have now confirmed with siRNA silencing that beta catenin is not required for SFRP2 induced endothelial tube formation. We also previously found that NFATc3 is activated in endothelial cells after SFRP2 treatment, and now confirm with shRNA silencing that NFATc3 is required for SFRP2 induced tube formation. This suggests that targeting NFAT with calcineurin inhibitors is a potential therapeutic approach to inhibit angiogenesis induced by both VEGF and SFRP2.

Several lines of evidence suggest that calcineurin inhibitors like tacrolimus might have efficacy against tumor growth. Tacrolimus has previously been shown to inhibit the growth of an NFAT overexpressing mouse leukemia xenograft *in vivo* [22] and the SVR angiosarcoma xenograft *in vivo* [5].Tacrolimus has not been tested for its ability to inhibit breast cancer in humans, however in a large series of patients receiving tacrolimus following liver transplantation the *de novo* incidence of breast cancer was found to be 1.9 times lower than matched controls from the SEER database [23]. This correlation has only been observed in specific patient populations but in light of our study demonstrating that the binding partner for tacrolimus, FKBP12, is expressed in the vasculature of human breast tumors, and that tacrolimus inhibits the growth rate of MMTV-neu transgenic breast carcinomas in mice, there is good rationale for prospectively investigating the clinical utility of tacrolimus in breast cancer treatment.

There are several calcineurin inhibitors that are FDA approved for the prevention of organ transplant rejection, although only tacrolimus and cyclosporine inhibit NFAT. Sirolimus (rapomycin) and everolimus (RAD001, a derivative of rapomycin) have the same intracellular target as tacrolimus, FKBP12, but unlike tacrolimus these drugs inhibit mTOR and have no effect on NFAT [24]. Because the safety profile of tacrolimus is better than cyclosporine, we chose to study tacrolimus for efficacy at inhibiting breast tumor growth.

The anticancer potential of calcineurin inhibitors like tacrolimus is likely not restricted to their ability to antagonize angiogenesis in the vasculature. Calcineurin/NFAT signaling cascades are important for proliferation and migration in a number of cell types. In this study we demonstrated that tacrolimus inhibited not only endothelial cell migration, but also breast tumor cell migration. Based on the critical importance of these modalities in cancer progression and maintenance it is not surprising that a growing number of studies have identified significant dysregulation of NFAT/calcineurin signaling in a variety of human tumors [25]. Increased NFAT/calcineurin activity has been observed in several human carcinomas including pancreatic [26], colon [27], and breast cell carcinoma [28]. In melanoma BRAF has been shown to activate NFAT to direct transcription of cyclooxygenase, a process which is also antagonized by tacrolimus [5]. Buchholz and colleagues have shown that about 70% of pancreatic carcinomas have elevated levels of nuclear NFATc1 compared to healthy pancreatic tissues. Using human-derived pancreatic carcinoma cell lines, they demonstrated that the nuclear localization of transcriptionally active NFATc1 is a calcineurin-dependent process that was inhibited by cyclosporine A. Treatment with cyclosporine A also inhibited *in vitro* cell cycle progression and anchorage-independent proliferation of the Panc1 cell line [26]. *In vitro* tacrolimus has been shown to inhibit hepatocellular carcinoma [29], [30], and prostate cancer proliferation [31]. Studies such as these further support the investigation of calcineurin inhibitors like tacrolimus in anti-cancer regimens.

In summary, we demonstrated that tacrolimus is able to inhibit *in vitro* tube formation stimulated by both SFRP2 and VEGF and inhibit the migration of endothelial and breast cancer cells. Tacrolimus may be particularly useful in the treatment of breast cancer as it attenuated breast tumor xenograft growth *in vivo*, and FKBP12 is expressed in the vasculature of human breast carcinomas. Given the utility of other antiangiogenic factors in breast cancer [32] and the previous observation of reduced breast cancer incidence following tacrolimus administration [23], there is ample rationale for examining the therapeutic potential of tacrolimus as part of an anti-breast cancer chemotherapeutic regimen.

## Supporting Information

Figure S1 Immunohistochemistry with antibodies to FKBP12 on paraffin embedded human breast carcinomas showed localization of FKBP12 to endothelium. Arrows point to vessels. Pictures taken at 200× magnification. A) Negative control showed no background staining. B) Mouse MMTV-neu tumor showed FKBP12 staining of vessels. C & D) Human breast tumors showed FKBP12 staining of vessels.
(DOC)

Figure S2 Full length blot/gels with molecular markers. A) Western blot results of siRNA to beta catenin shows beta catenin is present in 2H11 endothelial cells and sham-transfected 2H11 endothelial cells, but reduced in siRNA to beta catenin transfected 2H11 endothelial cells. B) Western blot results of ShRNA to SFRP2 in 2H11 endothelial cells showed increased NFATc3 protein in 2H11 endothelial cells stimulated with 7 nM SFRP2 compared to control 2H11 endothelial cells. This effect is abolished when tacrolimus is added. C) Western blot results of shRNA to NFATc3 showed NFATc3 protein is present in sham-transfected 2H11 endothelial cells, but reduced in shRNA to NFATc3 transfected 2H11 endothelial cells.
(DOC)

Figure S3 Pictures of endothelial cells in Matrigel tube formation assay. A) Sham transfected 2H11 endothelial cells stimulated with 7 nM mouse recombinant SFRP2 for 6 hours branch and form tube in Matrigel. B) ShRNA to NFATc3 transfected 2H11 cells stimulated with 7 nM mouse recombinant SFRP2 for 6 hours do not undergo tube formation in Matrigel.
(DOC)
