# Supplementary figures and images for: The Role of Calcineurin/NFAT in SFRP2 Induced Angiogenesis—A Rationale for Breast Cancer Treatment with the Calcineurin Inhibitor Tacrolimus
Source: PLoS One. 2011 Jun 3;6(6):e20412. doi: 10.1371/journal.pone.0020412 (PMC3108822; doi:10.1371/journal.pone.0020412)

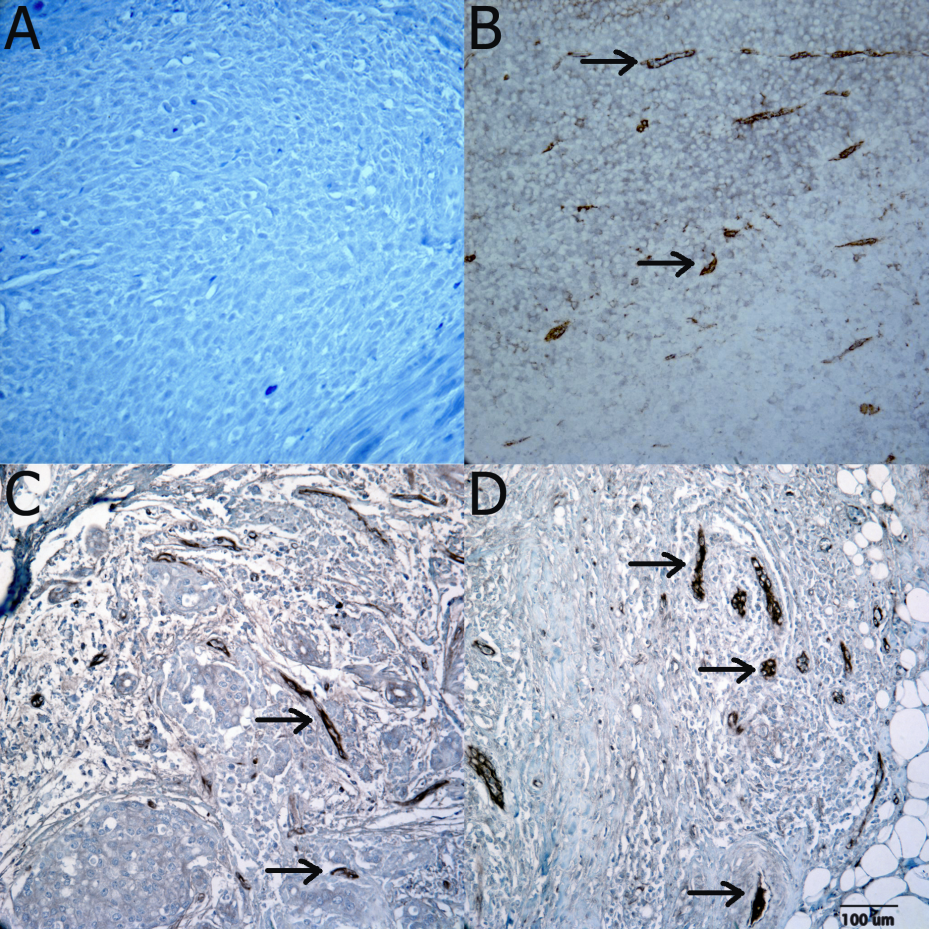

Supplement: Figure S1 — Immunohistochemistry with antibodies to FKBP12 on paraffin embedded human breast carcinomas showed localization of FKBP12 to endothelium. Arrows point to vessels. Pictures taken at 200× magnification. A) Negative control showed no background staining. B) Mouse MMTV-neu tumor showed FKBP12 staining of vessels. C & D) Human breast tumors showed FKBP12 staining of vessels. (DOC) [file pone.0020412.s001.doc]

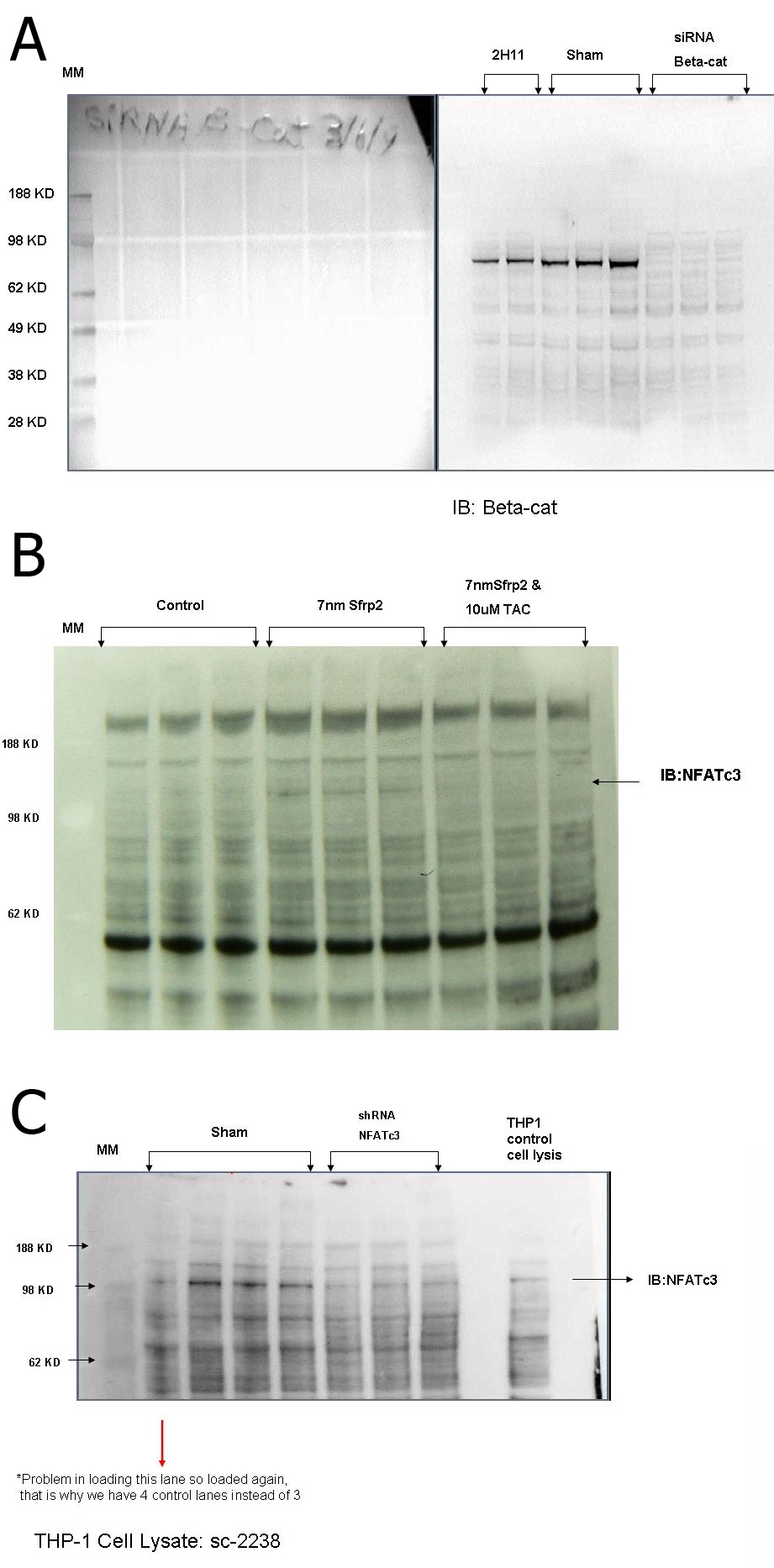

Supplement: Figure S2 — Full length blot/gels with molecular markers. A) Western blot results of siRNA to beta catenin shows beta catenin is present in 2H11 endothelial cells and sham-transfected 2H11 endothelial cells, but reduced in siRNA to beta catenin transfected 2H11 endothelial cells. B) Western blot results of ShRNA to SFRP2 in 2H11 endothelial cells showed increased NFATc3 protein in 2H11 endothelial cells stimulated with 7 nM SFRP2 compared to control 2H11 endothelial cells. This effect is abolished when tacrolimus is added. C) Western blot results of shRNA to NFATc3 showed NFATc3 protein is present in sham-transfected 2H11 endothelial cells, but reduced in shRNA to NFATc3 transfected 2H11 endothelial cells. (DOC) [file pone.0020412.s002.doc]

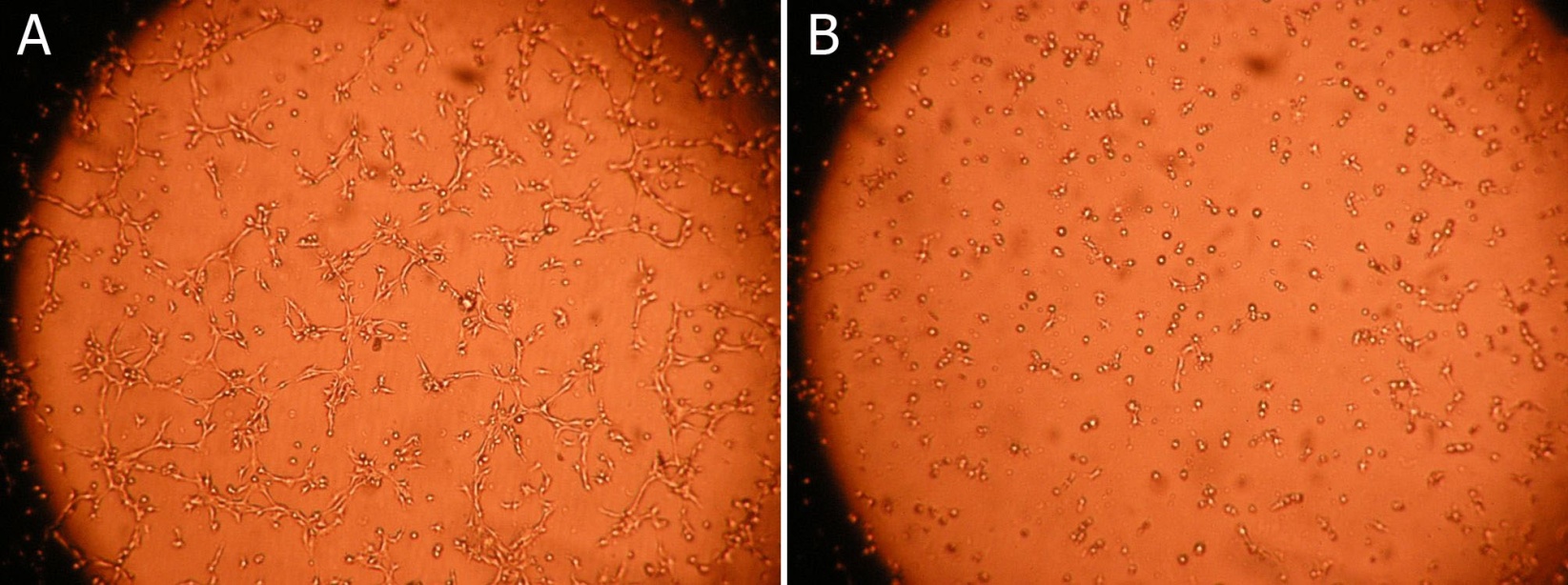

Supplement: Figure S3 — Pictures of endothelial cells in Matrigel tube formation assay. A) Sham transfected 2H11 endothelial cells stimulated with 7 nM mouse recombinant SFRP2 for 6 hours branch and form tube in Matrigel. B) ShRNA to NFATc3 transfected 2H11 cells stimulated with 7 nM mouse recombinant SFRP2 for 6 hours do not undergo tube formation in Matrigel. (DOC) [file pone.0020412.s003.doc]
